# Supplementary material for: Investigating and promoting health behaviors reactivity among Hong Kong older adults in the post-COVID-19 Era: An exploratory network analysis
Source: PLoS One. 2023 Nov 2;18(11):e0293512. doi: 10.1371/journal.pone.0293512 (PMC10621926; doi:10.1371/journal.pone.0293512)
Supplement: S1 File — (ZIP) [file pone.0293512.s001.zip › Supporting information/S1 Table. Centrality measures of the healthy lifestyle behaviour variables.docx]

S1 Table. Centrality measures of the healthy lifestyle behaviour variables

|  |  | Strength (Degree) | Closeness | Betweenness |
| --- | --- | --- | --- | --- |
| Sleeping Time | Before | 1.00 | 0.00081 | 0 |
|  | During | 1.04 | 0.00082 | 29 |
| Sleeping Quality | Before | 1.02 | 0.002 | 30 |
|  | During | 1.12 | 0.002 | 64 |
| Eating Habits | Before | 0.82 | 0.001 | 28 |
|  | During | 0.77 | 0.001 | 0 |
| Leisure Activities | Before | 1.20 | 0.002 | 60 |
|  | During | 1.09 | 0.002 | 0 |
| Social Network | Before | 1.28 | 0.002 | 88 |
|  | During | 0.86 | 0.002 | 12 |
| Bodily Pain | Before | 0.74 | 0.001 | 8 |
|  | During | 0.83 | 0.001 | 20 |
| Exercise Efficacy | Before | 1.09 | 0.002 | 48 |
|  | During | 1.22 | 0.002 | 42 |
| Resilience | Before | 1.22 | 0.002 | 14 |
|  | During | 1.12 | 0.002 | 0 |
