# Supplementary material for: Investigating and promoting health behaviors reactivity among Hong Kong older adults in the post-COVID-19 Era: An exploratory network analysis
Source: PLoS One. 2023 Nov 2;18(11):e0293512. doi: 10.1371/journal.pone.0293512 (PMC10621926; doi:10.1371/journal.pone.0293512)
Supplement: S1 File — (ZIP) [file pone.0293512.s001.zip › Supporting information/S2 Table. Edges Weight Matrix.docx]

S2 Table. Edges Weight Matrix

|  | 1 | 2 | 3 | 4 | 5 | 6 | 7 | 8 | 9 | 10 | 11 | 12 | 13 | 14 | 15 | 16 |
| --- | --- | --- | --- | --- | --- | --- | --- | --- | --- | --- | --- | --- | --- | --- | --- | --- |
| Sleeping_Time | 1 | 0.98 | -0.008 | 0 | -0.005 | 0 | 0 | -0.001 | 0.0016 | 0.0016 | -0.007 | 0 | 0 | 0 | 0 | 0.0002 |
| Sleeping_Time_COVID | 0.98 | 1 | -0.01 | -0.01 | 0 | 0 | 0.0029 | 0 | 0 | 0.009 | 0 | 0 | 0 | -0.006 | -0.009 | 0 |
| Sleeping_Quality | -0.008 | -0.010 | 1 | 0.80 | -0.006 | 0 | -0.023 | -0.006 | -0.05 | 0 | -0.010 | 0 | -0.036 | 0.057 | -0.008 | -0.005 |
| Sleeping_Quality_COVID | 0 | -0.02 | 0.80 | 1 | 0 | 0.01 | 0.029 | 0.004 | 0.024 | -0.0023 | 0 | 0.029 | 0.05 | -0.11 | -0.009 | -0.024 |
| Eating_Habits | -0.005 | 0 | -0.006 | 0 | 1 | 0.72 | 0.02 | -0.009 | 0 | -0.011 | 0 | -0.019 | -0.008 | 0.0056 | 0 | 0.007 |
| Eating_Habits_COVID | 0 | 0 | 0 | 0.01 | 0.71 | 1 | 0 | 0 | 0.01 | 0 | 0.002 | 0.01 | 0 | -0.008 | 0.004 | 0 |
| Leisure_Activity | 0 | 0.002 | -0.02 | 0.028 | 0.025 | 0 | 1 | 0.76 | 0.25 | -0.06 | -0.014 | 0 | 0.0043 | 0.0079 | 0 | -0.008 |
| Leisure_Activity_COVID | -0.001 | 0 | -0.0061 | 0.0045 | -0.0094 | 0 | 0.76 | 1 | -0.14 | 0.08 | 0 | 0.011 | 0 | 0.057 | 0 | -0.010 |
| Social_Network | 0.0016 | 0 | -0.04 | 0.02 | 0 | 0.01 | 0.25 | -0.14 | 1 | 0.59 | 0 | 0.023 | 0.087 | -0.036 | 0.038368 | -0.019 |
| Social_Network_COVID | 0.0016 | 0.0092 | 0 | -0.002 | -0.01 | 0 | -0.06 | 0.08 | 0.59 | 1 | 0.012 | -0.030 | -0.03 | 0 | 0.0075 | 0 |
| Bodily_Pain | -0.007 | 0 | -0.009 | 0 | 0 | 0.002 | -0.014 | 0 | 0 | 0.01 | 1 | 0.66 | 0 | -0.03 | 0 | 0 |
| Bodily_Pain_COVID | 0 | 0 | 0 | 0.029 | -0.02 | 0.01 | 0 | 0.01 | 0.02 | -0.03 | 0.67 | 1 | 0.0093 | 0 | 0.014 | -0.020 |
| Exercise_efficacy | 0 | 0 | -0.03 | 0.051842 | -0.008 | 0 | 0.0043 | 0 | 0.086 | -0.03 | 0 | 0.0093 | 1 | 0.65 | 0.167 | -0.034 |
| Exercise_efficacy_COVID | 0 | -0.007 | 0.057 | -0.11 | 0.005 | -0.008 | 0.0079 | 0.057 | -0.04 | 0 | -0.027 | 0 | 0.65 | 1 | -0.104 | 0.13 |
| Resilience | 0 | -0.0088 | -0.0079 | -0.009 | 0 | 0.0044 | 0 | 0 | 0.038 | 0.0075 | 0 | 0.014 | 0.17 | -0.10 | 1 | 0.86 |
| Resilience_COVID | 0.00026 | 0 | -0.0052 | -0.024 | 0.0070 | 0 | -0.008 | -0.0104 | -0.02 | 0 | 0 | -0.02 | -0.03 | 0.13 | 0.86 | 0 |
