# Supplementary material for: Investigating and promoting health behaviors reactivity among Hong Kong older adults in the post-COVID-19 Era: An exploratory network analysis
Source: PLoS One. 2023 Nov 2;18(11):e0293512. doi: 10.1371/journal.pone.0293512 (PMC10621926; doi:10.1371/journal.pone.0293512)
Supplement: S1 File — (ZIP) [file pone.0293512.s001.zip › Supporting information/S3 Table. Paths Summary Table.docx]

S3 Table. Paths Summary Table

| Timepoint |  | Direct Effect | Indirect Effect | Total Effect |
| --- | --- | --- | --- | --- |
| Before | Bodily Pain 🡪 Resilience | 0.39** | / | 0.39** |
|  | Sleeping Quality 🡪 leisure activities 🡪 resilience | / | 0.18** | 0.18** |
|  | Resilience 🡪 social network | 0.37** | / | 0.37** |
|  | Social network🡪 sleeping quality | 0.67** | / | 0.67** |
|  | Resilience 🡪 social network 🡪 sleeping quality | / | 0.25** | 0.25** |
|  | Bodily Pain 🡪 Exercise self-efficacy | -0.61** | / |  |
|  | Sleeping quality 🡪 Eating habits | 0.49** | / | 0.49** |
|  | Sleeping quality 🡪 Eating habits 🡪 eating habits during COVID-19 | / | 0.20** | 0.20** |
| During | Eating Habits 🡪 resilience | 0.6** | / | 0.6** |
|  | Social network🡪eating habits 🡪 resilience | / | 0.004** | 0.004** |
|  | Resilience 🡪 leisure activities | 0.31** | / | 0.31** |
|  | Sleeping Time 🡪 Eating Habits 🡪 Sleeping Quality | / | 0.032** | 0.032** |
|  | Sleeping quality 🡪 Sleeping Time | -0.43** | / | -0.43** |
|  | Bodily Pain 🡪 Exercise self-efficacy | 0.64** | / | 0.64** |
| ***p*<0.001 | | | | |
