# Supplementary figures and images for: Investigating and promoting health behaviors reactivity among Hong Kong older adults in the post-COVID-19 Era: An exploratory network analysis
Source: PLoS One. 2023 Nov 2;18(11):e0293512. doi: 10.1371/journal.pone.0293512 (PMC10621926; doi:10.1371/journal.pone.0293512)

S1 Figure. The Centrality Plot


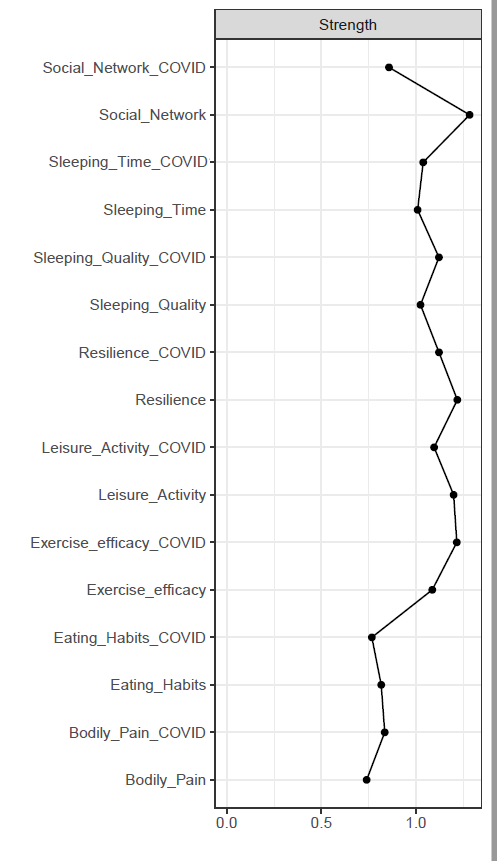

Supplement: S1 File — (ZIP) [file pone.0293512.s001.zip › Supporting information/S1 Figure. The Centrality Plot.docx]

S2 Figure. The Centrality Stability Plot


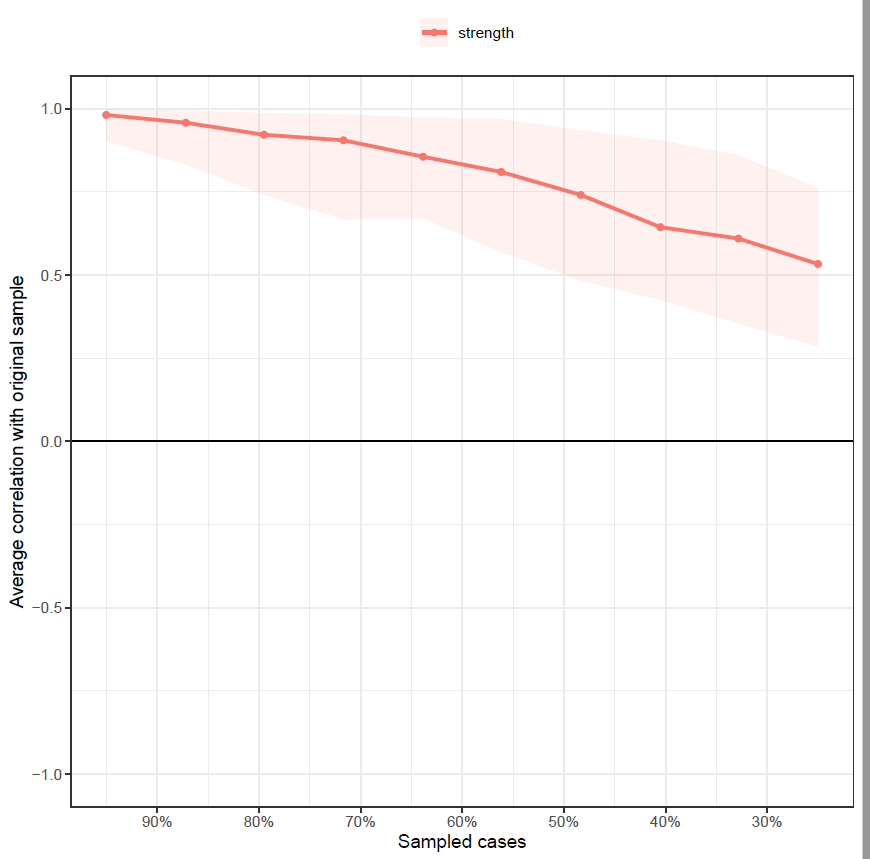

Supplement: S1 File — (ZIP) [file pone.0293512.s001.zip › Supporting information/S2 Figure. The Centrality Stability Plot.docx]

S3 Figure. Edge Stability


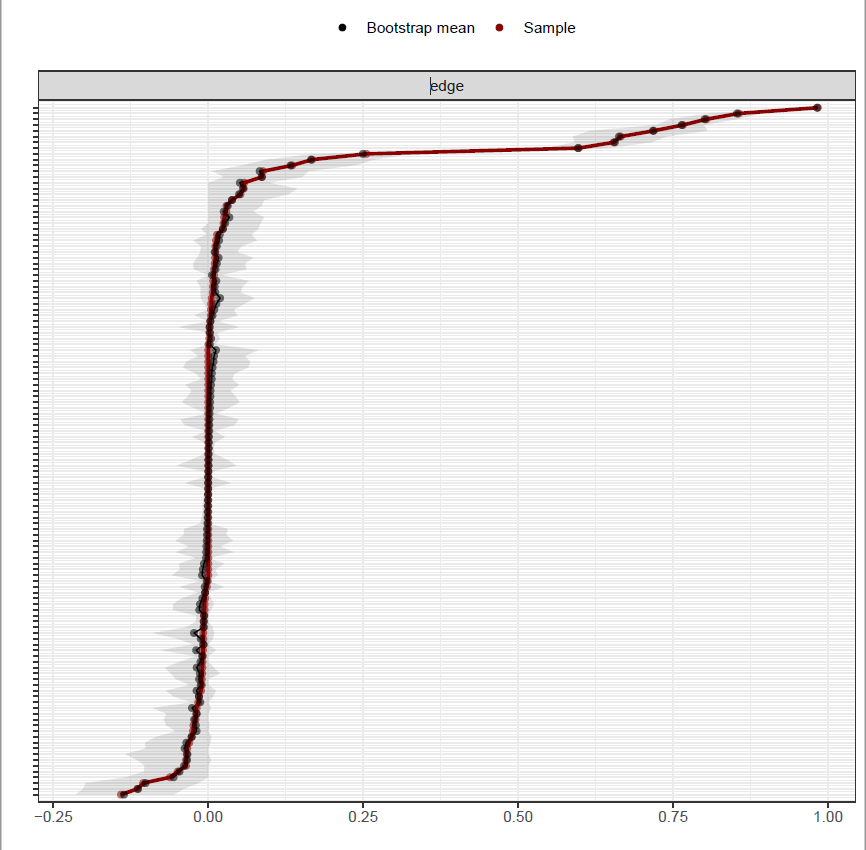

Supplement: S1 File — (ZIP) [file pone.0293512.s001.zip › Supporting information/S3 Figure. Edge Stability.docx]
