# Supplementary material for: Investigating and promoting health behaviors reactivity among Hong Kong older adults in the post-COVID-19 Era: An exploratory network analysis
Source: PLoS One. 2023 Nov 2;18(11):e0293512. doi: 10.1371/journal.pone.0293512 (PMC10621926; doi:10.1371/journal.pone.0293512)
Supplement: Attachment — Submitted filename: Reply to reviewer[1].docx [file pone.0293512.s002.docx]

Reply to reviewer

Reviewer #1: The methods and results should be SIMPLE PAST not past perfect or any other tense.

Thanks for your comments, the tense has been revised.

More citations and evidence are needed to support reliability, validity, psychometric properties, and factor structure of the measures and questionnaires.

Supported references have been added for the corresponding methods.

Too many abbreviations. Define them, and reduce them.

Those abbreviations were defined.

Fit data of various models to be presented in a table.

The authors do understand the reviewer’s concern, however, as there’s only one model to be shown, therefore, it was directly shown inside the manuscript.

What are * when p value is reported in the text of the results? Is it a typo?

The Typo has been revised.

The results may not hold for older old (80 yrs), low SES and high SES, and ethnic groups. Please test invariance by these factors.

The authors agreed with the reviewer’s concern. While, the study has included around 30% of older adults aged 80 or above, which is much higher than that of aged between 75-79, hence we believe the results could hold for all older adults age groups. In spite of that, the invariance model between age groups have been tried, yet the observations of each group is too small with lots of paths and indicators to perform the model invariance test.

Unfortunately, due to the focus on COVID-19 and the majority low monthly income of older adults, the social status and ethnicity of Hong Kong older adults were not involved in the telephone survey.

Reviewer #2: Revisions are required. Please address the following comments and questions.
1. Strengthen theoretical background. Elaborate in more depth. Connect theory with research design and discussion.
2. Literature review is on the brief side. Requires more discussion, analysis, and citations.

The authors well understood the suggestion offered by the reviewer. The authors have added more literature and citation regarding the importance and phenomenon of Chinese older adults’ physical activity and other healthy lifestyle changes and effect during COVID-19. However, the current manuscript is being treated as a deductive explorative means to investigate the relationship between the healthy lifestyle indicators in a data-driven approach, yet not theory-driven. The social networking method is considered as a tool to demonstrate the relationships between the indicators but not aiming to present it as a theory or conceptual framework. Therefore, the authors have managed to add the definition of the healthy lifestyle behavior, and the importance of older adults’ perceived exercise efficacy and resilience.

3. List the objectives in a separate section;
4. Include a separate research gap section;

The research objectives and research gap were separated as suggested.

5. List the hypotheses;

Hypotheses are not applicable to explorative social network analysis with the research outcome as data-driven; and with no research in regards to COVID-19 has been done on Hong Kong older adults’ perceived exercise efficacy and resilience.

6. Line 128: Would COVID-19 be considered as a traumatic event?

Thanks for your concerns. Similar to previous pandemics, such as SARS, COVID is a large-scale and highly contagious virus, and the city experienced a lockdown during the epidemic. some literature also highlights that it is responsible for some post-traumatic symptoms and some emotional reactions such as anger, anxiety, and helplessness. Hong Kong has experienced five waves of the epidemic and many elderly people have died as a result, so we believe that COVID is considered to be traumatic to some extent. (Bridgland et al., 2021).

7. Provide reliability and validity figures for the psychometric instruments;

Some indexes for representing reliability and validity were added.

8. Add response rate; “has proved”
9. Line 328: tone down

The new sentence: “Most of the literature claimed the effect of COVID-19 on older adults’ physical inactivity”

10. Line 342: add citations

Reference had been added.

11. Discussion, implications, and future research directions sections: Elaborate in more depth.

Please see the implication and future direction added in the discussion.

12. Lines 47 and 399: tone down “representative of the Hong Kong community.”

The new sentence: “it may be representative of the Hong Kong community”

13. Line 35: “tele survey” -> telephone survey

Revised
14. “sleeping quality” -> sleep quality

Revised

Reference

Bridgland, V. M., Moeck, E. K., Green, D. M., Swain, T. L., Nayda, D. M., Matson, L. A., ... & Takarangi, M. K. (2021). Why the COVID-19 pandemic is a traumatic stressor. *PloS one*, *16*(1), e0240146.
